# Synthesis and Biological Characterization of the New Glycolipid Lactose Undecylenate (URB1418)

**DOI:** 10.3390/ph15040456

**Published:** 2022-04-08

**Authors:** Michele Verboni, Serena Benedetti, Raffaella Campana, Francesco Palma, Lucia Potenza, Maurizio Sisti, Andrea Duranti, Simone Lucarini

**Affiliations:** Department of Biomolecular Sciences, University of Urbino Carlo Bo, 61029 Urbino, PU, Italy; m.verboni@campus.uniurb.it (M.V.); serena.benedetti@uniurb.it (S.B.); raffaella.campana@uniurb.it (R.C.); francesco.palma@uniurb.it (F.P.); lucia.potenza@uniurb.it (L.P.); maurizio.sisti@uniurb.it (M.S.); simone.lucarini@uniurb.it (S.L.)

**Keywords:** glycolipids, lactose monoesters, enzymatic synthesis, sugar-based surfactants, fatty acids, minimum inhibitory concentration (MIC), antifungal agents

## Abstract

As a follow-up to our previous studies on glycolipid surfactants, a new molecule, that is lactose 6′-*O*-undecylenate (URB1418), was investigated. To this end, a practical synthesis and studies aimed at exploring its specific properties were carried out. URB1418 showed antifungal activities against *Trichophyton rubrum* F2 and *Candida albicans* ATCC 10231 (MIC 512 μg/mL) and no significant antibacterial activity against *Staphylococcus aureus* and *Pseudomonas aeruginosa*. At the same time, it presented anti-inflammatory properties, as documented by the dose-dependent reduction in LPS-induced NO release in RAW 264.7 cells, while a low antioxidant capacity in the range of concentrations tested (EC_50_ > 200 µM) was also observed. Moreover, URB1418 offers the advantage of being more stable than the reference polyunsaturated lactose esters and of being synthesized using a “green” procedure, involving an enzymatic method, high yield and low manufacturing cost. For all these reasons and the absence of toxicity (HaCaT cells), the new glycolipid presented herein could be considered an interesting compound for applications in various fields.

## 1. Introduction

Glycolipids are amphiphilic compounds characterized by a sugar hydrophilic polar head (usually mono- or disaccharides) and an apolar tail composed of a saturated or unsaturated fatty chain. The interest in these molecules, classified as surfactants due to their ability to lower interfacial tension, is related to their increasingly widespread use in various industrial sectors, such as pharmaceuticals, food and cosmetics, due to the favorable properties. In fact, they have an excellent biocompatibility profile, being non-irritating, non-toxic and biodegradable [1]. A further advantage of glycolipid surfactants is given by the ease with which they can be obtained, even on a large scale, from renewable sources and inexpensive raw materials [2]. As a demonstration of this, for their realization, although traditional synthetic chemistry is still used in some cases (e.g., reactions with aromatic or polyunsaturated fatty acids), synthetic methodologies based on enzymes are increasing more and more because they offer the advantage of soft reaction conditions and allow high yields with regioselectivity. In this field of research, in recent years, the studies on glycolipids with lactose as their sugar portion have increased [3,4]. In particular, lactose-based esters with saturated and unsaturated fatty acid, alkyl aromatic and aromatic tails were reported together with their emulsifying, biocompatibility, safety, permeability-enhancing, antimicrobial and antibiofilm properties [5,6,7,8,9]. Specifically, mono-unsaturated lactose esters, such as lactose oleate, lactose nervonate and lactose palmitoleate, displayed a combination of macromolecular absorption enhancing activity and antimicrobial properties together with a low toxicity against human cell lines, indicating their potential use as absorption enhancers and/or preservatives [5,7]. On the other hand, similar enhancing properties were shown by the saturated lactose esters reported in Ref [6], however, with a toxicity profile on Calu-3 and Caco-2 human cell lines that increases with the chain length (C16 > C14 > C12) [6]. Moreover, lactose laurate (C12) enhanced permeability, likely via tight junction modulation in rat intestinal tissue [8]. Conversely, alkyl aromatic and aromatic derivatives, in particular lactose biphenylacetate, showed a safer toxicity profile compared to saturated lactose esters and a relatively remarkable antibacterial and antibiofilm activity [9].

**Figure 1:**
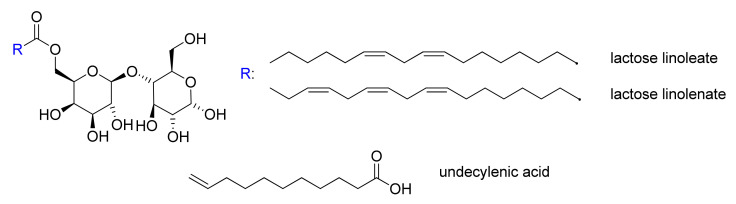
Structural formulas of lactose linoleate, lactose linolenate and undecylenic acid.

Very recently, lactose linoleate and lactose linolenate (Figure 1) have been studied in vitro for their cytocompatibility, anti-inflammatory, antioxidant and antibacterial activity. In addition, lactose linoleate in combination with chitosan showed a wound closure above 90% in male Wistar albino rats linked with the restoration of the Wnt/β-catenin signaling, demonstrating potential interest as agents for wound care [10].

Furthermore, glycolipids are potentially usable in invasive fungal infections, which represent a serious problem for human health and are common in immunocompromised patients subjected to anticancer therapy, long-term corticosteroid treatments or organ transplant, as well as immunosuppressive infections. In addition, fungi are responsible for infections of the skin and mucous membranes with a greater incidence compared to invasive infection. The development of antifungal drugs faces off with the fact that fungi are eukaryotes as their hosts; thus, the potential targets may also be found in human cells, therefore posing a substantial risk of toxicity. Due to this scenario, the acid from which the aliphatic chain of the drug discussed herein (lactose undecylenate) is derived, namely undecylenic acid (UA, Figure 1), is considered an ancient remedy against fungal skin infection [11], even if there is no detailed information regarding its mechanism of action.

Moreover, it should also be considered that, in the protective response against microbial invasion, another scenario, such as the inflammatory process, plays a key role. It is characterized by complex interactions between resident and infiltrating cells, soluble mediators and extracellular matrix molecules. A controlled inflammation leads to the removal of harmful stimuli and re-establishes a physiological state; however, if the acute form fails to retain the pro-inflammatory stimulus, chronic inflammation and tissue damage may occur. Therefore, inflammation is a key therapeutic target for pharmacological interventions, and the development of novel approaches is fundamental for a better management of acute and chronic inflammatory conditions [12,13,14]. In this context, surfactants have recently gained attention for their potential role as immunomodulatory and anti-inflammatory agents because of their ability to regulate the immune system and the inflammatory response both in in vitro and in vivo models [15,16].

The study presented here follows those previously carried out on sugar esters of various characteristics, as previously described [5,6,7,8,9,17], and aims to contribute to the development of new glycolipid molecules, potentially useful in various fields of research and industry. We then focus our attention on the synthesis and characterization of a new lactose ester named 6′-*O*-undec-10-enoyl-4-*O*-(β-d-galactopyranosyl)-d-glucopyranose (lactose undecylenate, URB1418) through a “green” synthetic procedure and an exploration of its antifungal, antibacterial, anti-inflammatory, antioxidant and safety properties.

## 2. Results and Discussion

### 2.1. Chemistry

**Scheme 1:**
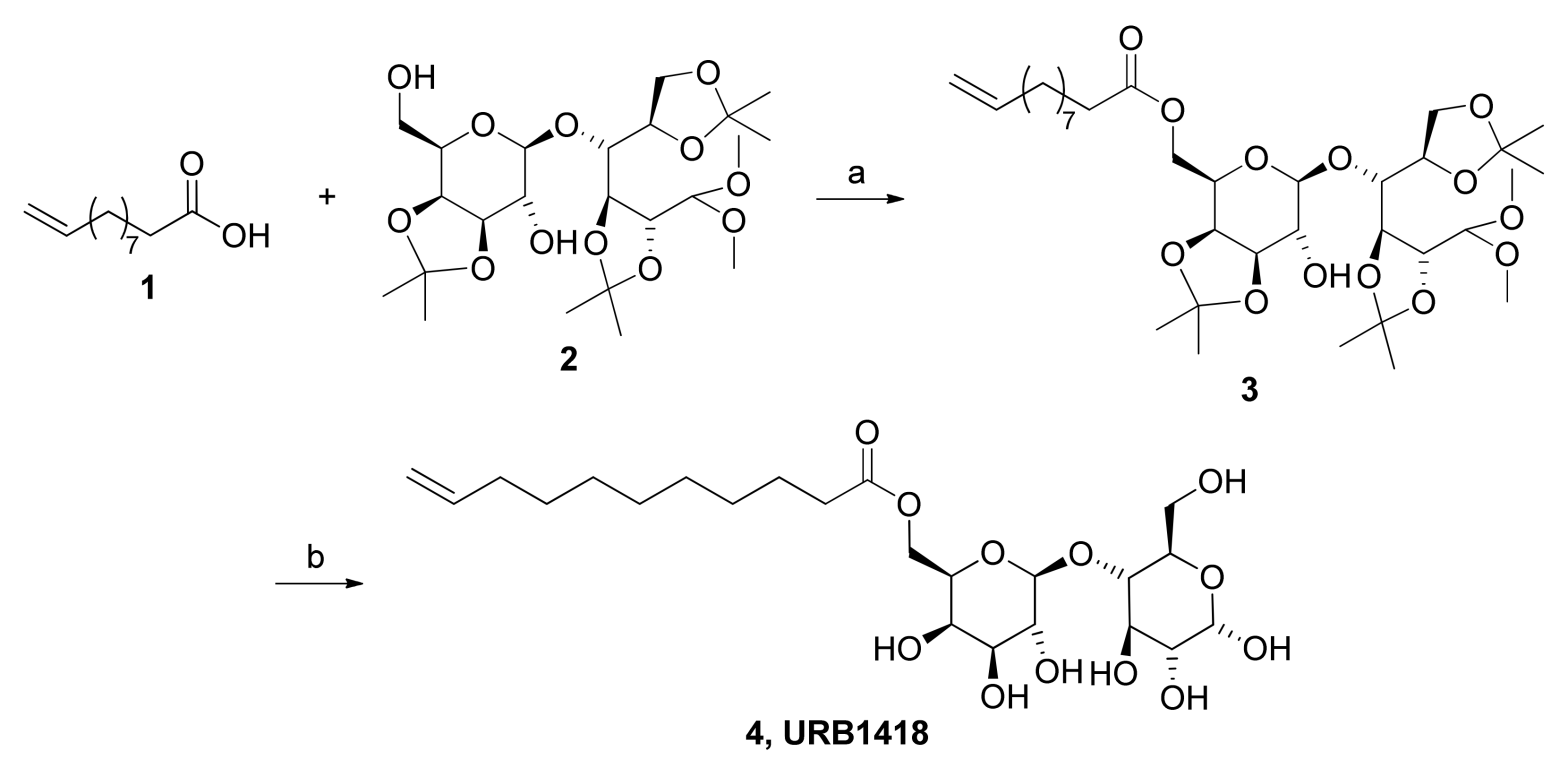
Reagents and conditions: (**a**) toluene, 75 °C, 12 h; (**b**) HBF_4_^.^Et_2_O/H_2_O/CH_3_CN, 0 °C, 45 h.

Lactose undecylenate was synthesized through a two-step protocol according to a procedure for the synthesis of lactose-based esters [5]. Firstly, the coupling of the undecylenic acid (**1**) with 4-*O*-(3′,4′-*O*-isopropylidene-β-d-galactopyranosyl)-2,3:5,6-di-*O*-isopropylidene-1,1-di-*O*-methyl-d-glucopyranose (lactose tetra acetal, LTA, **2**) using the catalyzing lipase Lipozyme^®^ in dry toluene furnished LTA ester **3** (LTA undecylenate). Subsequent deprotection of the latter by HBF_4_ led to a precipitate, which was filtered and recrystallized to obtain the final product **4** (URB1418) (Scheme 1).

The described synthetic procedure offers various advantages in comparison to classic chemical routes in terms of the use of green chemistry principles, manufacturing costs and yields.

### 2.2. Antifungal and Antibacterial Activity

All the antifungals available at present have various drawbacks in terms of toxicity, spectrum of activity, safety, pharmacokinetic properties and the occurrence of resistant strains [18,19]. Consequently, the need for new antifungals, as well as new antibiotics, has led to great efforts to search for and develop new drugs with different mechanisms of action to avoid cross-resistance and/or cross-toxicities. In this direction, the antifungal as well as the antibacterial potential of URB1418 was examined here. Data are summarized in Table 1 and Table 2 and indicate an antifungal activity comparable to that of undecylenic acid (UA).

With regard to URB1418, minimum inhibitory concentration (MIC) values of 512 µg/mL were observed in the case of *T. rubrum* F2 and *C. albicans*, while no activity was evidenced against the other fungal strains (MIC > 1024 µg/mL). As expected, UA showed wide antifungal activity with MIC ranging from 1024 to 128 µg/mL. More recently, the antifungal activities of lactose linoleate and lactose linolenate against the same fungal species were reported. Lactose linoleate showed lower MIC values compared to URB1418 against *T. mentagrophytes* F6, *E. floccosum* F12 and *C. albicans* ATCC 10231 (256, 128 and 256 µg/mL, respectively). Similarly, lactose linolenate was active against *T. mentagrophytes* F6 (MIC = 256 µg/mL) and *E. floccosum* F12 (MIC = 128 µg/mL) [10].

Conversely, URB1418 was not active against the tested bacterial strains. In particular, MIC values greater than 1024 µg/mL were observed against all the *S. aureus* strains (*S. aureus* HCS026, *S. aureus* 2/5, *S. aureus* 28/10, *S. aureus* 18/9 and *S. aureus* ATCC 43387), resulting as ineffective also against the two MRSA strains (*S. aureus* HCS002 and *S. aureus* ATCC 43300) and *P. aeruginosa* strains. Similarly to URB1418, lactose linoleate and lactose linolenate showed no or low activity against the same *S. aureus* and *P. aeruginosa* strains, with MIC values of 1024 or > 1024 µg/mL [10]. On the other hand, other monounsaturated lactose esters, such as lactose palmitoleate and lactose nervonate, were able to inhibit the growth of Gram-positive and Gram-negative bacteria, showing MIC values between 64 and 128 µg/mL [5]. Similarly, sugar-based esters with different hydrophobic chains and polar heads displayed wide antibacterial activity (MIC in the range 128–256 µg/mL) [8].

Our data are partially in agreement with those reported by other researchers who evidenced antifungal activity of similar undecylenic esters against *C. albicans* as well as against several pathogenic microorganisms, including *S. aureus* and *P. aeruginosa* spp. [20]. In particular, Shi et al. [21] reported that *C. albicans* UA reduced the hypha-to-yeast ratio, causing the deformation of cell surface and inhibiting hyphal formation, adhesion ability, mitochondrial activity, cell proliferation and transcriptional regulation of the cell membrane’s virulent factors [22,23]. However, the mechanism of action of UA is not completely known, but it was hypothesized that the fungal cell membrane is one of the drug targets of fatty acids, suggesting that enzymes or metabolites associated with lipid homeostasis may be affected by this molecule. On the other hand, these observations were supported by other researchers on the antimicrobial activity of sucrose fatty acid monoesters [24] or different sugar-based fatty acid esters and glycolipids [25,26]. With regard to the available antifungals, zinc undecylenate is a commercial topical agent for the treatment of skin infections, such as athlete’s foot, and relieves the itching, burning and irritation associated with the skin condition. Due to its bifunctional properties, undecylenate is also indicated as a linker to conjugate other biomolecules, such as proteins. It is proposed that UA exerts antimicrobial actions interacting with nonspecific components in the cell membrane [23]. Moreover, to our knowledge, no data are yet available on UA sugar ester antifungal activity, while some information on undecylenic aliphatic esters or other sugar-based fatty acid esters is well reported in the literature [20,26]. For all these reasons, a comparison between our data and those in the literature is difficult, also considering the difference in the tested compounds and microbiological assays utilized.

### 2.3. Anti-Inflammatory Properties

**Figure 2:**
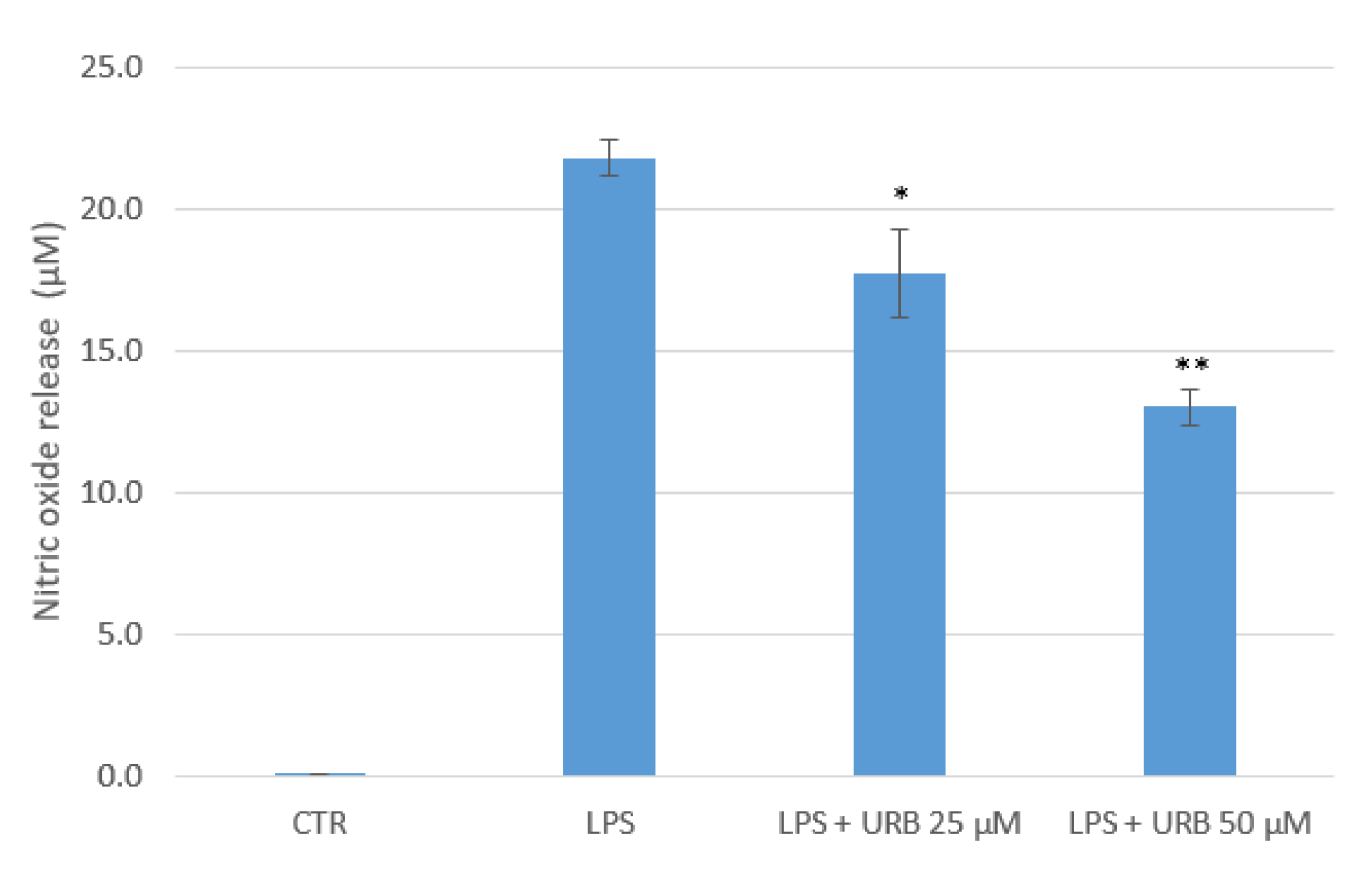
Assessment of the anti-inflammatory properties of URB1418 (URB) against LPS-induced NO production in RAW 264.7 cells by the reagent Griess. * *p* < 0.05 vs. LPS, ** *p* < 0.01 vs. LPS.

The administration of lipopolysaccharide (LPS) to RAW 264.7 cells (murine macrophages) led to nitric oxide (NO) production and a release in the culture medium, while NO could not be quantified in untreated control cells (Figure 2). When cells were pre-treated for 2 h with URB1418 25 µM and 50 µM, a significant dose-dependent reduction in LPS-induced NO release was observed (−19% and −40%, respectively).

**Figure 3:**
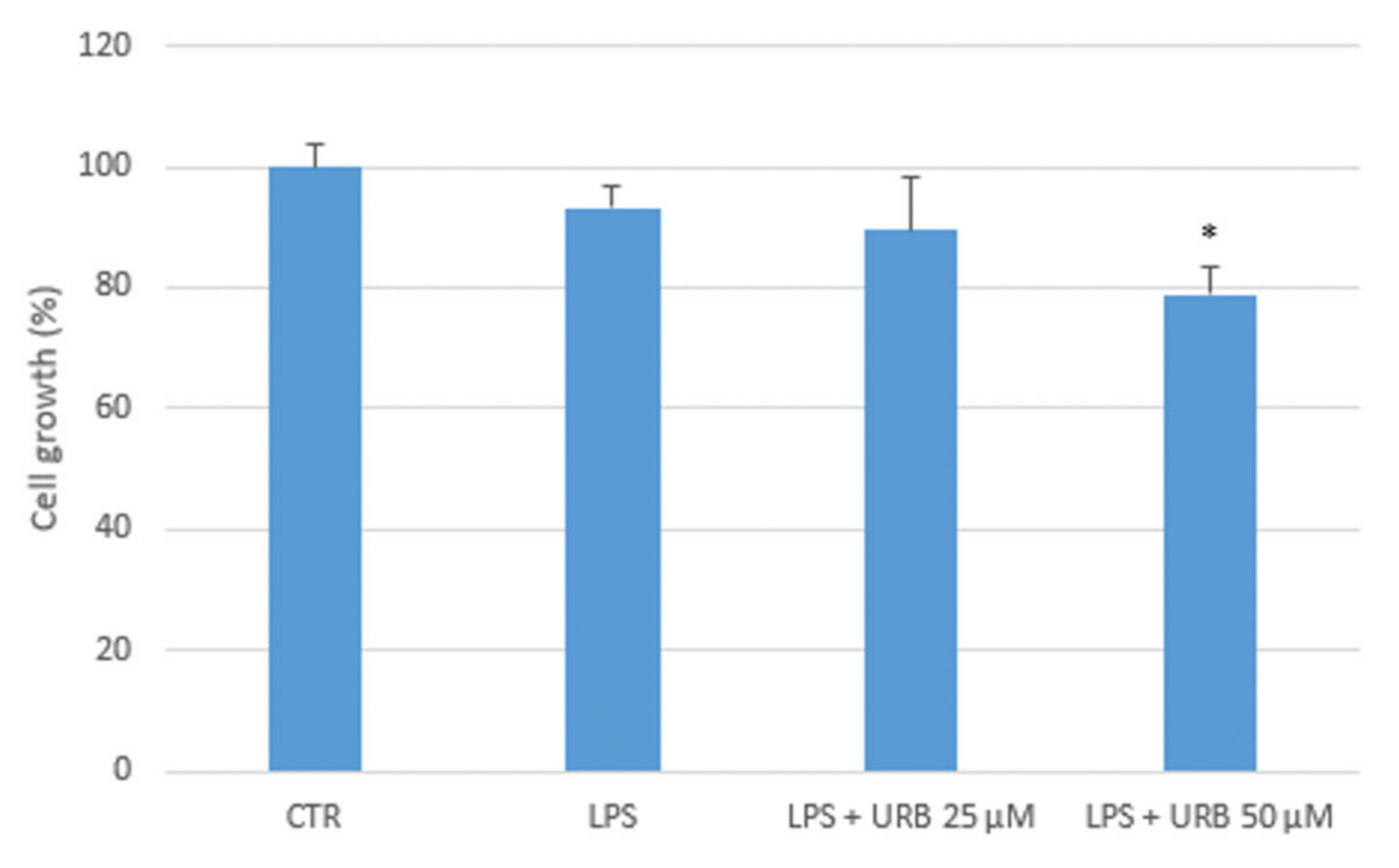
Evaluation of cell growth after URB1418 (URB) administration to RAW 264.7 cells stimulated with LPS (SRB test). * *p* < 0.05 vs. control.

The evaluation of RAW 264.7 proliferation in LPS-stimulated cells revealed only a slight decrease in cell growth by URB1418 50 µM (Figure 3). In both situations, data are expressed as mean ± SD of three independent experiments. NO is recognized as a mediator and regulator of the inflammatory response. In fact, the exposure of inflammatory cells and tissue cells to microbial products, such bacterial LPS, induces the expression of inducible nitric oxide synthase (iNOS), which in turn enhances NO production [27]. The ability of URB1418 to reduce NO release by LPS-activated macrophages suggests potential anti-inflammatory effects of the surfactant against microbial infections. Notably, these results agree with those previously obtained with other types of surfactants, especially of natural origin (biosurfactants). Indeed, it was demonstrated that both sophorolipid and surfactin, employed at concentrations comparable to those of URB1418, have the ability to decrease the production of NO and other inflammatory mediators in RAW 264.7 cells stimulated by LPS [28,29], thereby sustaining their potential therapeutic role as immunomodulators [15]. With reference to lactose-based surfactants, we recently reported that lactose linoleate and lactose linolenate at the concentration of 50 µM significantly reduced NO release in LPS-activated cells by 60% and 50%, respectively [10]. Other recent studies reported the synthesis and biological evaluation of disaccharide-based anionic amphiphiles on the modulation of pro-inflammatory responses.

These glycolipids were derived from the nonreducing β,α-1,1′-linked diglucosamine scaffold and are antagonists of pro-inflammatory signaling mediated by Toll-like receptor 4 (TLR4)/myeloid differentiation-2 (MD-2) complex [30,31,32]. However, very little is known regarding the anti-inflammatory activity of lactose-based surfactant, and to our knowledge, this is the first study other than that with lactose linoleate and lactose linolenate [10].

### 2.4. Radical Scavenging Properties

**Figure 4:**
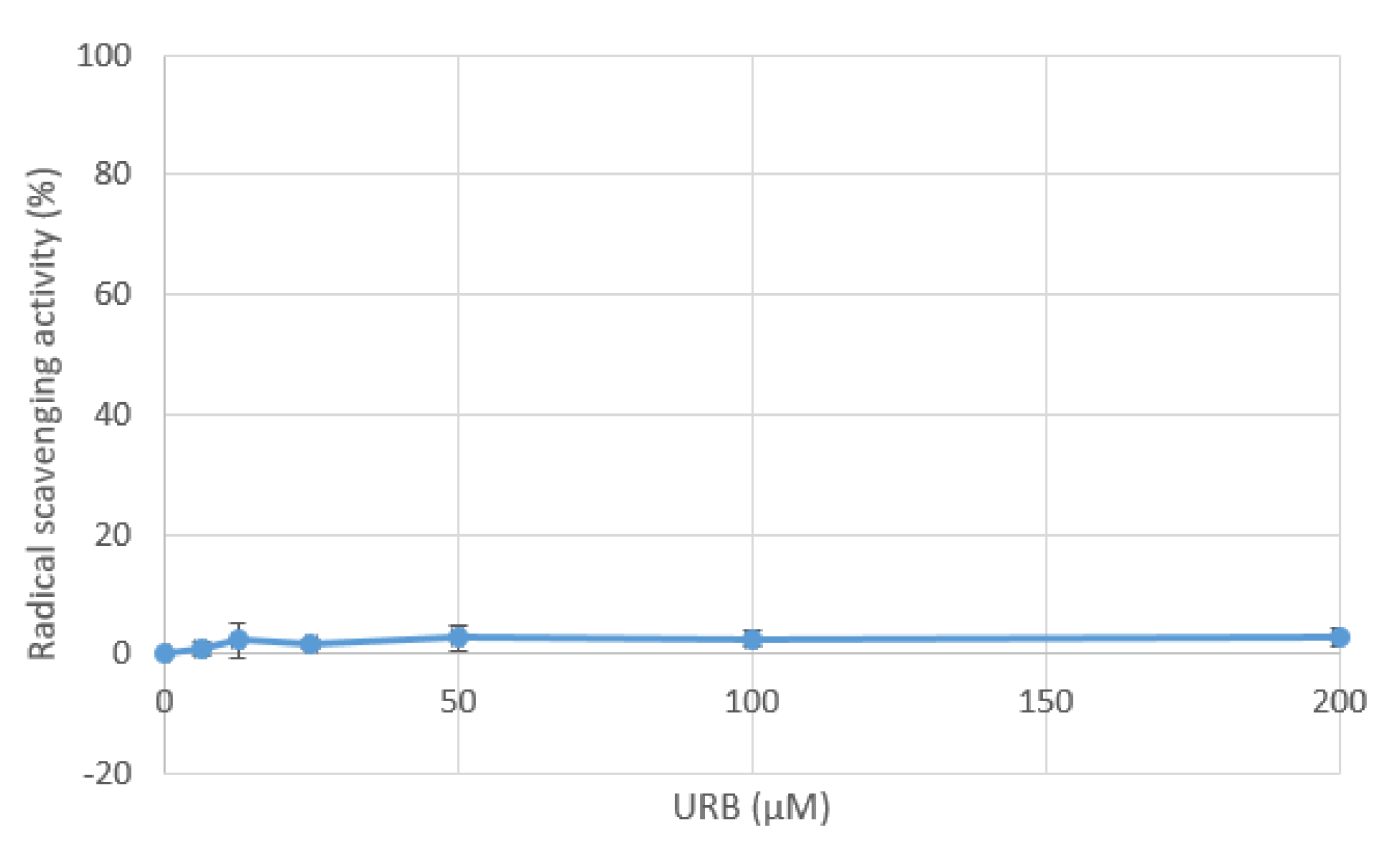
Evaluation of the radical scavenging activity of URB1418 (URB) by DPPH assay.

The 2,2-diphenyl-1-picrylhydrazyl hydrate (DPPH) assay evidenced that URB1418 did not show appreciable radical scavenging activity within the range of concentrations tested (EC_50_ > 200 µM) (Figure 4).

**Figure 5:**
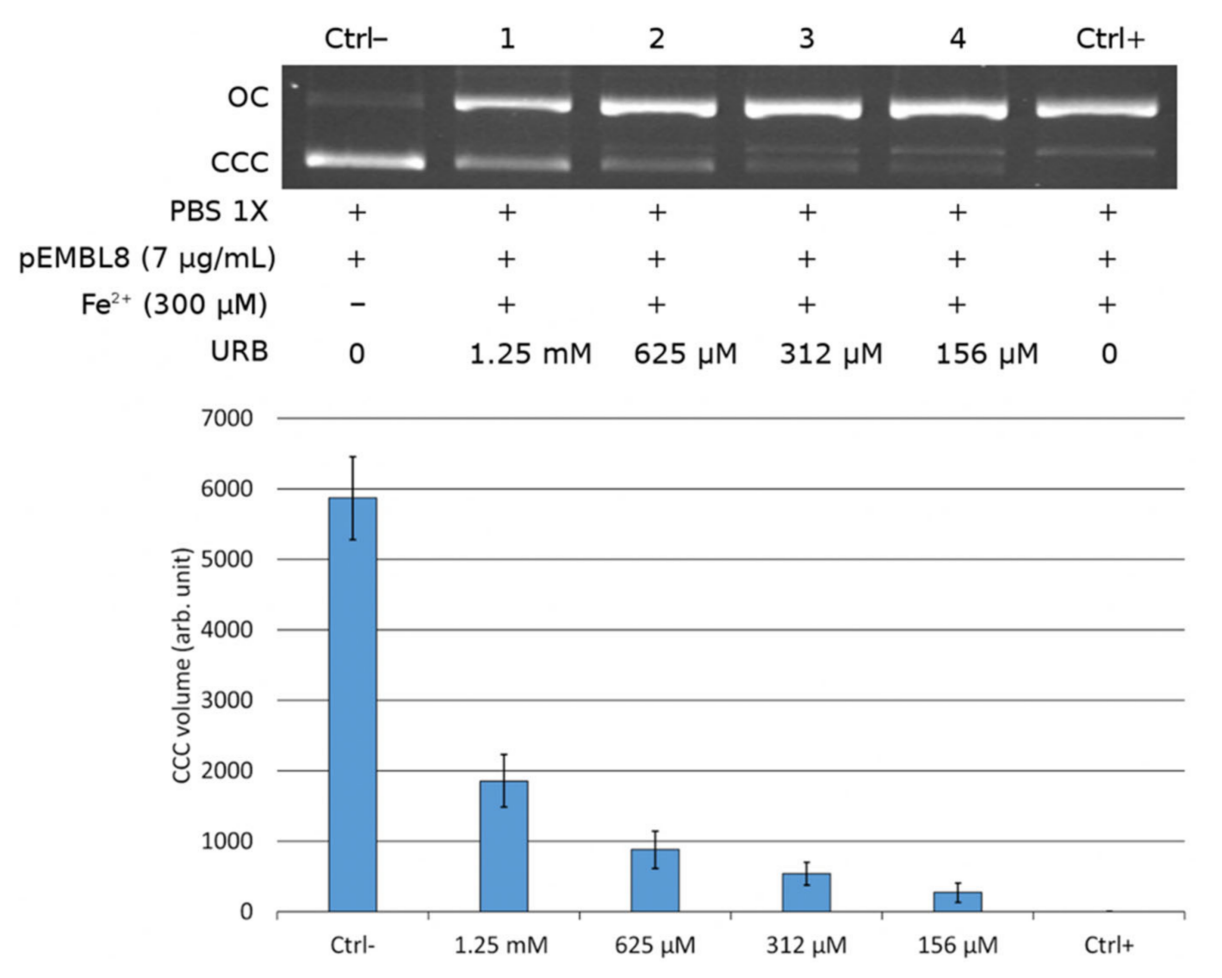
Evaluation of the protective activity of URB1418 (URB) vs. oxidative DNA damage by DNA nicking assay.

The DNA nicking assay also did not evidence protective activity vs. oxidative DNA damage (Figure 5); the highest URB1418 concentration tested (1.25 mM) was not able to raise the EC_50_ value, established at >3 mM. In both situations, data are expressed as mean ± SD of three independent experiments.

The DPPH assay has been widely used to investigate the antioxidant properties of surfactants [33,34,35,36]. In general, these compounds have lower radical scavenging activities than the reference antioxidant molecules, such as ascorbic acid. In particular, they show scavenger abilities at concentrations ranging from 1 to 10 mg/mL, which are far higher than those herein tested for URB1418 (up to 200 µM, corresponding approximately to 0.1 mg/mL). In this context, we previously demonstrated that lactose linoleate and lactose linolenate had EC_50_ values equal to 407 ± 11 μM and 396 ± 8 μM, respectively [10].

With reference to the DNA nicking assay, which uses ferrous ions to generate free radicals, we recently applied this test to lactose linoleate and lactose linolenate, showing EC_50_ values of 510 ± 80 μM and 600 ± 70 μM, respectively [10]. Other antioxidant assays investigating the ferrous ion chelating ability of surfactants demonstrate that their maximum activity is observed at concentrations > 1 mg/mL [34,36]. Accordingly, the EC_50_ value of URB1418 is established at >3 mM, corresponding to 1.5 mg/mL.

### 2.5. Evaluation of the Antioxidant Properties

**Figure 6:**
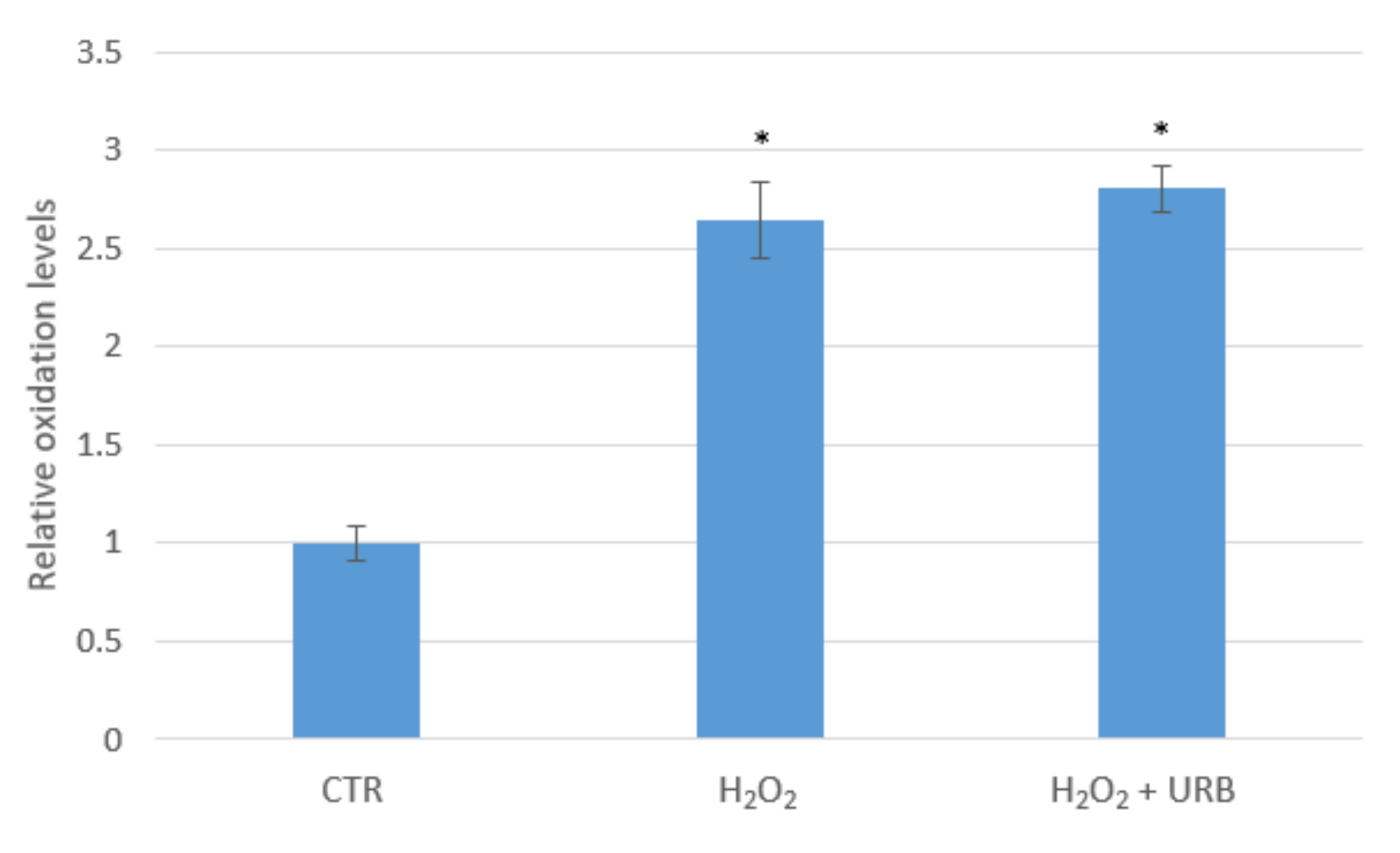
Assessment of the antioxidant capacity of URB1418 (URB) against H_2_O_2_-induced oxidation in HaCaT cells by the probe DCFH-DA. * *p* < 0.05 vs. control.

The administration of H_2_O_2_ to cultured human keratinocyte (HaCaT cells) led to a significant increase in 2′,7′-dichlorofluorescein (DCF) fluorescence emission, indicating increased intracellular oxidation levels as compared to untreated cells (Figure 6).

When cells were pre-treated for 2 h with URB1418 100 µM, no reduction in H_2_O_2_-induced oxidation was observed, suggesting that URB1418 did not show antioxidant protective capacity against H_2_O_2_. The same finding was evidenced also in the case of lactose linoleate and lactose linolenate at 100 µM [10]. These results appear to be in agreement with those obtained by DPPH and DNA nicking assays, confirming that URB1418 shows no antioxidant properties within the range of concentrations tested (up to 200 µM, corresponding to 0.1 mg/mL). Accordingly, surfactant antioxidant activities were recorded at concentrations ranging from 1 to 10 mg/mL [33,34,35,36].

### 2.6. Evaluation of the Cytotoxic Effects

**Figure 7:**
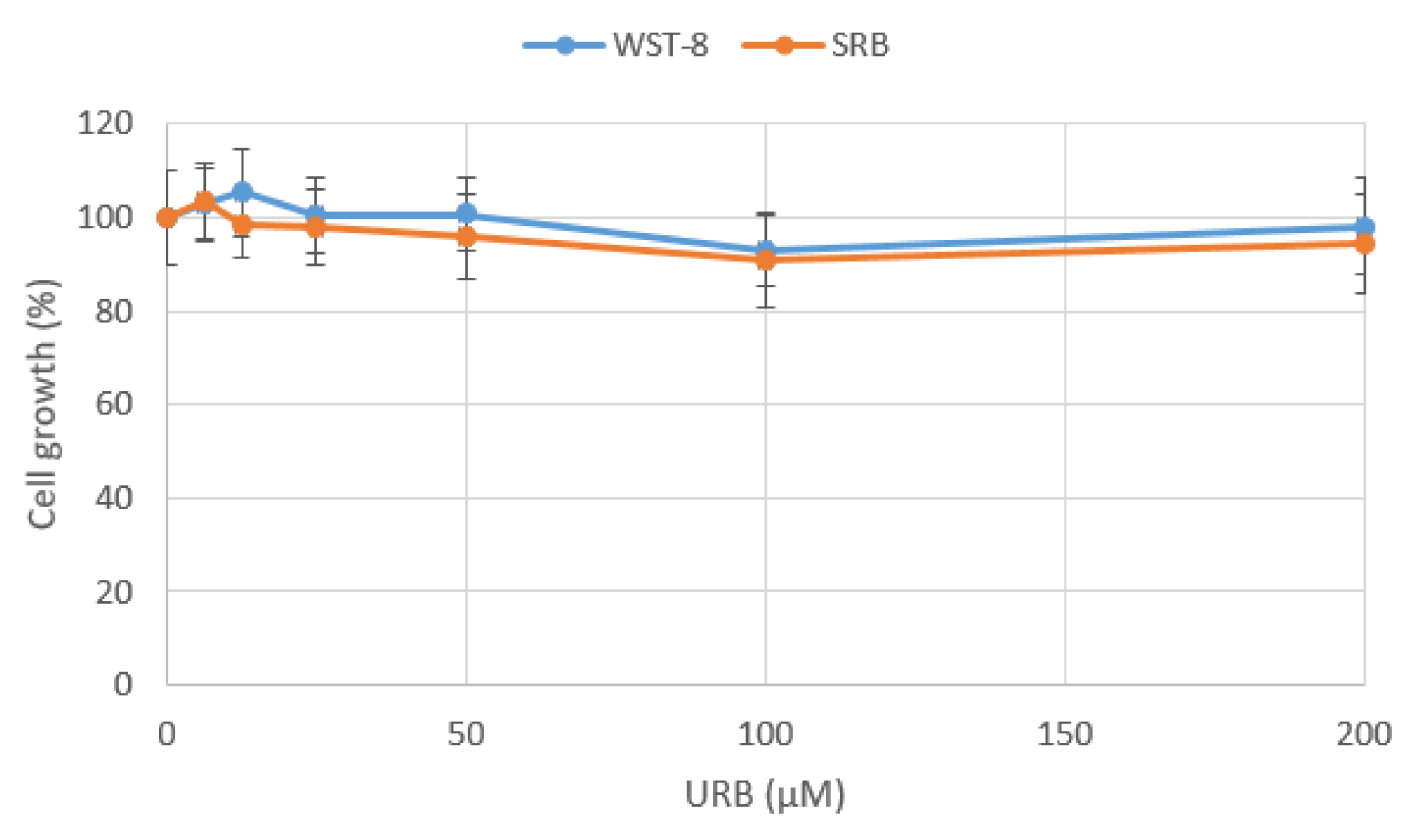
HaCaT cell growth after URB1418 (URB) administration evaluated by WST-8 and SRB assays.

Potential cytotoxic effects of URB1418 administration were analyzed in HaCaT cells both by WST-8 and SRB assays, leading to comparable results and demonstrating that URB1418 was not cytotoxic in the range of concentrations tested (Figure 7). Data are expressed as mean ± SD of three independent experiments. In comparison to lactose linoleate and lactose linolenate, a significant reduction in cell growth by both molecules at the concentration 200 µM was observed [10].

## 3. Materials and Methods

### 3.1. Chemicals

Lactose monohydrate was purchased from Carlo Erba (Milan, Italy). Undecylenic acid, Lipozyme^®^ (immobilized lipase from Mucor miehei), tetrafluoroboric acid diethyl ether complex (HBF_4_^.^Et_2_O), acetonitrile (CH_3_CN), ethyl acetate (EtOAc), methanol (MeOH), toluene, and deuterated solvents chloroform (CDCl_3_) and dimethylsulfoxide (DMSO-*d6*) were purchased from Sigma-Aldrich (Milan, Italy). Prior to use, CH_3_CN was dried with molecular sieves with an effective pore diameter of 4 Å. The structures of the compounds were unambiguously assessed by MS, ^1^H NMR and ^13^C NMR. ESI-MS spectra were recorded with a Waters Micromass ZQ spectrometer in a negative or positive mode using a nebulizing nitrogen gas at 400 L/min and a temperature of 250 °C, cone flow 40 mL/min, capillary 3.5 kV and cone voltage 60 V; only molecular ions [M − H]^−^, [M + NH_4_]^+^, [M + Na]^+^ or [M + HCOO]^−^ are given. ^1^H NMR and ^13^C NMR spectra were recorded on a Bruker AC 400 or 101 spectrometer, respectively, and analyzed using the TopSpin 1.3 software package. Chemical shifts were measured by using the central peak of the solvent. The original ESI-MS, ^1^H, COSY and ^13^C spectra of **3** and URB1418 are reported in the Supplementary Materials. Column chromatography purifications were performed under “flash” conditions using Merck 230–400 mesh silica gel. TLC was carried out on Merck silica gel 60 F254 plates, which were visualized by exposure to ultraviolet light and to an aqueous solution of ceric ammonium molybdate.

### 3.2. Synthesis of 6′-O-Undec-10-enoyl-4-O-(3′,4′-O-isopropylidene-β-d-galactopyranosyl)-2,3:5,6-di-O-isopropylidene-1,1-di-O-methyl-d-glucopyranose (3, LTA Undecylenate)

Lipozyme^®^ (0.200 g) was added to a solution of undecylenic acid (**1**) (0.184 g, 1 mmol) and 4-*O*-(3′,4′-*O*-isopropylidene-β-d-galactopyranosyl)-2,3:5,6-di-*O*-isopropylidene-1,1-di-*O*-methyl-d-glucopyranose (lactose tetra acetate, LTA) [37] (**2**) (0.508 g, 1 mmol) in dry toluene (0.63 mL) at 30 °C [5,38]. The mixture was stirred at 75 °C for 12 h, cooled, diluted with acetone, then filtered, and the filtrate was concentrated. The purification of the residue by column chromatography (cyclohexane/EtOAc 8:2, rf: 0.11) gave **3** (LTA undecylenate) as a yellow pale oil. Yield: 79% (0.401 g). MS(ESI): 692 [M + NH_4_]^+^, 719 [M + HCOO]^−^. ^1^H NMR (400 MHz, CDCl_3_): δ = 1.26–1.31 [m, 8H, (CH_2_)_4_], 1.32–1.35 (m, 8H, 2 CH_3_ and *CH_2_*CH_2_CH=CH_2_) 1.38 (s, 3H, CH_3_), 1.39 (s, 3H, CH_3_), 1.49 (s, 3H, CH_3_), 1.51 (s, 3H, CH_3_), 1.58–1.64 (m, 2H, C*H_2_*CH_2_C=O), 2.00–2.06 (m, 2H, C*H_2_*CH=CH_2_), 2.32 (t, 2H, *J* = 7.5 Hz, CH_2_CH*_2_*C=O), 3.42 (s, 3H, OCH_3_), 3.43 (s, 3H, OCH_3_), 3.56 (dd, 1H, J_H2′-H3′_ = 7.0 Hz, *J*_H2′-H1′_ = 8.0 Hz, H^2′^), 3.90 (dd, 1H, *J*_H3-H4_ = 1.5 Hz, *J*_H3-H2_ = 7.5 Hz, H^3^), 3.94 (ddd, 1H, *J*_1_ = 2.0 Hz, *J*_2_ = 5.0 Hz, *J_3_* = 7.0 Hz), 4.01 (dd, 1H, *J*_1_ = 7.0 Hz, *J*_2_ = 9.0 Hz), 4.04–4.10 (m, 2H), 4.11 (dd, 1H, *J*_1_ = 2.0 Hz, *J*_2_ = 5.5 Hz), 4.16 (dd, 1H, *J*_1_ = 6.5 Hz, *J*_2_ = 9.0 Hz), 4.25–4.30 (m, 2H), 4.35 (dd, 1H, *J*_1_ = 5.0 Hz, *J*_2_ = 11.5 Hz), 4.37 (d, 1H, *J*_H1-H2_ = 6.0 Hz, H^1^), 4.42 (d, 1H, *J*_H1′-H2′_ = 8.0 Hz, H^1′^), 4.45 (dd, 1H, *J*_H2-H1_ = 6.0 Hz, *J*_H2-H3_ = 7.5 Hz, H^2^), 4.92 (dddd, 1H, *J*_gem_ ≅ *J*_1_ = 1.5 Hz*, J*_2_ = 4.0 Hz, *J*_cis_ = 10.0 Hz, HC*H*=CH), 4.98 (dddd, 1H, *J*_gem_ ≅ *J*_1_ ≅ *J*_2_ = 1.5 Hz, *J*_trans_ = 17.0 Hz*, H*CH=CH), 5.80 (dddd, 1H, *J*_1_ ≅ *J*_2_ = 7.0 Hz, *J*_cis_ = 10.0 Hz, *J*_trans_ = 17.0 Hz, HCH=C*H*) ppm. ^13^C NMR (100 MHz, CDCl_3_): δ = 24.6, 25.0, 25.8, 26.4, 26.5, 27.4, 28.2, 29.0, 29.17, 29.28, 29.34, 29.4, 33.9, 34.2, 53.3, 56.2, 63.2, 64.8, 71.5, 73.4, 74.3, 75.2, 76.5, 78.0, 78.1, 79.1, 103.8, 105.2, 108.4, 110.4, 110.5, 114.3 (CH=*C*H_2_), 139.2 (*C*H=CH_2_), 173.6 (C=O) ppm.

### 3.3. Synthesis of 6′-O-Undec-10-enoyl-4-O-(β-d-galactopyranosyl)-d-glucopyranose (4, Lactose Undecylenate, URB1418)

**3** (0.401 g, 0.59 mmol) was dissolved in HBF_4_^.^Et_2_O/H_2_O/CH_3_CN (4.7 mL, 1:5:500), and the mixture was stirred at 0 °C for 5 h. The white solid was then filtered, washed with CH_3_CN and dried. The purification by recrystallization from CH_3_OH gave **4** as white solid (rf: 0.45, CH_3_CN/H_2_O 95:5). Yield: 37% (0.110 g). Mp: 141–144 °C. MS(ESI): 507 [M − H]^−^, 526 [M + NH_4_]^+^, 531 [M + Na]^+^, 553 [M + HCOO]^−^. ^1^H NMR (400 MHz, DMSO-*d*_6_): δ = 1.21–1.28 [m, 8H, (CH_2_)_4_], 1.32–1.35 (m, 2H, C*H_2_*CH_2_CH=CH_2_), 1.47–1.56 (m, 2H, C*H_2_*CH_2_C=O), 1.97–2.04 (m, 2H, C*H_2_*CH=CH_2_), 2.30 (t, 2H, *J* = 7.5 Hz, CH_2_C*H_2_*C=O), 3.17 (ddd, 1H, *J*_H2–H1_ = 4.0 Hz, *J*_H2–OH2_ = 7.0 Hz, *J*_H2–H3_ = 9.5 Hz, H^2^), 3.27 (dd, 1H, *J*_H4–H3_ ≅ *J*_H4–H5_ = 9.5 Hz, H^4^), 3.32–3.38 (m, 2H, H^2′^, H^3′^), 3.56 (dd, 1H, *J*_H3–H2_ ≅ *J*_H3–H4_ = 9.5 Hz, H^3^), 3.61–3.66 (m, 3H, H6a, H6b, H^4′^), 3.68–3.75 (m, 2H, H5, H^5′^), 4.08 (dd, 1H, *J*_H6a′–H5_ = 4.0 Hz, *J*_H6a′–H6b′_ = 11.5 Hz, H^6a′^), 4.16 (dd, 1H, *J*_H6b′–H5′_ = 8.0 Hz, *J*_H6b′–H6a′_ = 11.5 Hz, H^6b′^), 4.20–4.26 (m, 2H, H1′, OH^3^), 4.47 (dd, 1H, *J*_OH6–H6a_ ≅ *J*_OH6–H6b_ = 6.0 Hz, OH^6^), 4.60 (d, 1H, *J*_OH2–H2_ = 7.0 Hz, OH^2^), 4.82 (d, 1H, *J*_OH4′–H4′_ = 6.5 Hz, OH^4′^), 4.87 (d, 1H, *J*_OH2′–H2′_ = 5.0 Hz, OH^2′^), 4.89 (dd, 1H, *J*_H1–OH1_ = 4.5 Hz, *J*_H1–H2_ = 4.0 Hz, H^1^), 4.93 (1H, dddd, *J*_cis_ = 10.0 Hz, *J*_2_ = 4.0 Hz, *J*_gem_ ≅ *J*_1_ = 1.5 Hz, HC*H*=CH), 4.99 (1H, dddd, *J*_trans_ = 17.0 Hz, *J*_gem_ ≅ *J*_1_ ≅ *J*_2_ = 1.5 Hz, *H*CH=CH), 5.79 (dddd, 1H, *J*_1_ ≅ *J*_2_ = 7.0 Hz, *J*_cis_ = 10.0 Hz, *J*_trans_ = 17.0 Hz, HCH=C*H*), 6.37 (d, 1H, *J*_OH1–H1_ = 4.5 Hz, OH^1^) ppm. ^13^C NMR (100 MHz, DMSO-*d*_6_): δ = 24.8, 28.7, 28.9 (2C), 29.13, 29.147, 33.6 (*C*HCH=CH_2_), 33.8 (*C*H_2_C=O), 60.9 (C6), 63.8 (C6′), 68.7 (C4′), 70.2 (C2), 70.7 (C2′), 71.7 (C3), 72.6 (C5), 72.9 (C3′), 73.3 (C5′), 81.5 (C4), 92.5 (C1), 104.0 (C1′), 115.1 (CH=*C*H_2_), 139.3 (*C*H=CH_2_), 173.4 (C=O) ppm.

### 3.4. Microbes and Culture Conditions

Pathogenic filamentous dermatophytes (collection of Pharmacology and Hygiene Section, Department of Biomolecular Sciences, University of Urbino) (*T. mentagrophytes* F6, *T. rubrum* F2, *T. violaceum* F11, *E. floccosum* F12) and the reference strain *C. albicans* ATCC 10231 were included. The dermatophytes were maintained on potato dextrose agar (PDA) (VWR) at 35 °C for 7 days, while *C. albicans* at 37 °C for 24 h.

In addition, five human clinical isolates of *S. aureus* (*S. aureus* HCS026, *S. aureus* 2/5, *S. aureus* 28/10, *S. aureus* 18/9, *S. aureus* HCS002 methicillin-resistant MRSA), as well as the clinical isolate *P. aeruginosa* C86, belonging to the strain collection of the above-mentioned Pharmacology and Hygiene Section, were used. The reference strains, *S. aureus* ATCC 43387, *S. aureus* ATCC 43300 (MRSA) and *P. aeruginosa* ATCC 27583 were also added. All the *S. aureus* strains were cultured in tryptone soy agar (TSA) (VWR, Milan, Italy) at 37 °C for 24 h, while *Pseudomonas* strains were grown in Cetrimide agar (VWR) at the same culture conditions. All the strains were stored at −80 °C in Nutrient broth (VWR) supplemented with 20% glycerol.

### 3.5. Minimum Inhibitory Concentration

MIC values were determined following the standard microdilution method (Clinical and Laboratory Standards Institute CLSI, 2017). Filamentous fungi and yeast suspensions were prepared according to CLSI M38-A and CLSI M27-A protocol, respectively. At first, the compound was dissolved (5 mg/mL) in biological grade DMSO (Sigma-Aldrich, Milan, Italy) to obtain the concentrated stock solution. Preliminary tests were carried out to exclude the possible microbicide activity of the used solvent; in any case, the volume of DMSO never exceeded 5 % (*v*/*v*) of the final total volume.

With regard to the filamentous fungi, spores were collected from PDA plate by adding 2 mL of sterile 0.85% saline solution with 0.05% Tween 80; the surface was scraped with a sterile cotton swab, the suspension was transferred to a new sterile tube and left at room temperature for 5 min to allow the hyphal fragments sedimentation. The upper suspension was then vigorously vortexed for 15 s and adjusted to an optical density of OD 530 nm between 0.09 and 0.4, corresponding to about 10^6^ spores/mL. In the case of *C. albicans*, the suspension was adjusted to a turbidity of 0.12 (about 10^7^ cfu/mL). Successively, 100 μL of each fungal or yeast suspensions was diluted 1:50 or 1:1000, respectively, in standard Roswell Park Memorial Institute (RPMI) 1640 medium (Sigma-Aldrich, Milan, Italy) and inoculated into 96-well plates together with the appropriate volumes of each test compound as described above. Additionally, two rows were left for positive control growth and negative control (medium only), and UA (from 1024 to 16 µg/mL) was used as internal control. Plates with dermatophytes were incubated at 35 °C, those containing *C. albicans* at 37 °C; all the plates were examined after 48 h of incubation. (MIC is defined as the lowest drug concentration able to inhibit the visible growth of microorganism in comparison with the untreated control sample). In addition, the turbidity of the 96-well plate was also assessed by spectrophotometer Multiskan EX (OD 530 nm) (Thermo Scientific, Waltham MA, USA).

For bacteria, one colony of each strain was incubated in 10 mL of tryptone soy broth (TSB) (VWR) at 37 °C for 18 h. The bacterial suspensions were adjusted to about 10^6^ CFU/mL (OD _610nm_ 0.13–0.15) in Mueller Hinton Broth II (MHB II) (VWR), and 100 µL was added to wells of the 96-well plate together with the appropriate volumes of the test solution (from 1024 to 16 µg/mL). Two rows were left as positive and negative controls inoculating bacteria alone and MHB II alone, respectively. MIC was defined as the lowest concentration of compound able to inhibit bacterial growth after 24 h of incubation at 37 °C.

### 3.6. DPPH Assays

The antioxidant capacity of URB1418 was first evaluated in a cell-free system by the DPPH radical scavenging assay, as previously described [39]. URB1418 was resuspended in DMSO at the concentration 100 mM and then diluted in ethanol (EtOH) (tested concentrations 6.25–200 µM). DPPH (100 µM, Sigma-Aldrich, Milan, Italy) was prepared in EtOH. The scavenger effect was expressed as % = [(OD 517 nm control) − (OD 517 nm sample/OD 517 nm control)] × 100. The concentration necessary to achieve a 50% antioxidant effect (EC_50_) was then calculated.

### 3.7. DNA Nicking Assays

The DNA nicking assay was employed to evaluate the protective effect of URB1418 (from 156 µM to 1.25 mM) against DNA oxidative damage. The test uses ferrous ions and dioxygen (Fe^2+^ + O_2_) to generate DNA strand breaks by free radicals. The system consisted of supercoiled pEMBL8 plasmid DNA [40]. The disappearance of the supercoiled form of the plasmid was assessed on an ethidium bromide-stained agarose gel electrophoresis followed by the quantitation by Gel Doc 2000 and Quantity One software (Bio-Rad). The EC_50_ value was calculated determining the concentration of the compound that protects half of the supercoiled plasmid.

### 3.8. Cytotoxicity Assays

HaCaT cells (immortalized human keratinocytes) were used to investigate URB1418 cytotoxicity in vitro. They were purchased from CLS-Cell Lines Service GmbH (Eppelheim, Germany). Cells (5 × 10^3^/well) were seeded in 96-well plates and incubated for 2 h with URB1418 (6.25–200 µM). The test compound was then removed and fresh medium added. After 24 h of incubation, cell growth was analyzed by both water-soluble tetrazolium (WST)-8 and SRB assays, which estimate the metabolic activity of the cells and their protein content, respectively [41,42]. Color development was monitored in a multi-well plate reader (BioRad Laboratories, Hercules, CA, USA) and data expressed as cell growth (%) vs. untreated control cells.

### 3.9. DCFH-DA Assay

The antioxidant properties of URB1418 in HaCaT cells were analyzed by 2′,7′-dichlorofluorescin diacetate (DCFH-DA, Sigma-Aldrich, Milan, Italy), which turns to highly fluorescent DCF after oxidation [43]. Cells (1 × 10^4^/well) were seeded in black 96-well plates and incubated for 2 h with URB1418 (100 µM). The test compound was then removed and DCFH-DA (5 μM) added to each well for 30 min at 37 °C. After excess probe removal, cells were oxidized with hydrogen peroxide (H_2_O_2_, 100 μM) for 30 min, and fluorescence emission (ex/em 485/520 nm) was measured in the multi-well plate reader FluoStar Optima (BMG Labtech, Ortenberg, Germany). Data were expressed as relative oxidation vs. non-oxidized cells.

### 3.10. Nitric Oxide Detection

The anti-inflammatory properties of URB1418 were evaluated in RAW 264.7 cells (murine macrophages) stimulated by LPS (Sigma-Aldrich, Milan, Italy). Cells (3 × 10^4^/well) were seeded in 96-well plates and treated for 2 h with URB1418 (25 and 50 µM). The test compound was then removed, and cells were incubated with 1 μg/mL LPS for 24 h. Thereafter, NO levels were determined in the medium by Griess reagent (Sigma-Aldrich, Milan, Italy) [44]. Absorbance was measured at 570 nm using a plate reader (BioRad Laboratories, Hercules, CA, USA). The SRB test was also performed in the same 96-well plate to evaluate RAW 264.7 cell growth after LPS treatment.

### 3.11. Statistical Analysis

Comparisons between multiple means were performed via ANOVA followed by Tukey’s post hoc test. Significance was set at *p* < 0.05. GraphPad Prism 6.0 (GraphPad Software, Inc., San Diego, CA, USA) was used for statistical analysis.

## 4. Conclusions

The sugar ester lactose undecylenate (URB1418) was obtained by applying a very advantageous synthetic methodology involving a “green” chemistry process. It is more stable than lactose linoleate and lactose linolenate, presents a weak antifungal activity and could offer some appreciable anti-inflammatory properties in vitro, even though it does not show significant antibacterial and antioxidant capacities.

The observed anti-inflammatory activity of URB1418 in LPS-activated macrophages suggests that the surfactant might be helpful in controlling the inflammatory response in fungal and microbial infections [45] and might have therapeutic potential as an immunomodulatory agent, as recently suggested for some biosurfactants [15,16].

With regard to microbiological aspects, URB1418 possess mostly antifungal rather than antibacterial activity. However, in the future, the investigation on the effect of this new compound could be extended to other important pathogen fungal strains (such as *Microsporum* spp., *Fusarium* spp. and *Aspergillus* spp.) to better define its activity and another potential field of application.

In fact, URB1418 did not display appreciable radical scavenging activity within the range of the tested concentrations, which were, however, far lower than the doses used in similar antioxidant assays with other surfactants [33,34,35,36].

Moreover, the new glycolipid herein studied is to be considered a safe drug, as toxicity was not revealed in the tests carried out.

Finally, it is noteworthy that URB1418 could be considered a potential prodrug of UA. However, its real application is often impaired by the oily nature, low solubility, unpleasant taste and odor, and a tendency to irritate [46]. Therefore, once administered, the prodrug could be a tool to avoid the problems mentioned above and to enhance the properties of the UA [21]. Due to these considerations, detailed in-depth studies regarding the mechanism of action of UA and the URB1418 prodrug scenario will still be needed and will furnish interesting information concerning the role of the glycolipid surfactant described in this study.

## Figures and Tables

**Table 1:** Antifungal activity of URB1418 assessed against filamentous fungi and *Candida albicans* strains. UA was used as internal control.

	MICs (µg/mL)	
	URB1418	UA
*Trichophyton mentagrophytes* F6	>1024	512
*Trichophyton rubrum* F2	512	256
*Trichophyton violaceum* F11	>1024	512
*Epidermophyton floccosum* F12	>1024	256
*C. albicans* ATCC 10231	512	128

**Table 2:** Antibacterial activity of URB1418 against *Staphylococcus aureus* and *Pseudomonas aeruginosa* strains.

	MICs (µg/mL) URB1418
*S. aureus* HCS026	>1024
*S. aureus* HCS002 (MRSA)	>1024
*S. aureus* 2/5	>1024
*S. aureus* 28/10	>1024
*S. aureus* 18/9	>1024
*S. aureus* MRSA ATCC 43300	>1024
*S. aureus* ATCC 43387	>1024
*P. aeruginosa* C86	>1024
*P. aeruginosa* ATCC 27583	>1024

## Data Availability

Data is contained within article and Supplementary Files.

## Supplementary Materials

The following are available online at https://www.mdpi.com/article/10.3390/ph15040456/s1, Figure S1: ^1^H NMR Spectra of LTA Undecylenate, Figure S2: ^1^H-^1^H COSY NMR Spectra of LTA Undecylenate, Figure S3: ^13^C NMR Spectra of LTA Undecylenate, Figure S4: MS(ESI) Spectra of LTA Undecylenate, Figure S5: ^1^H NMR Spectra of URB1418, Figure S6: ^1^H-^1^H COSY NMR Spectra of URB1418, Figure S7: ^13^C NMR Spectra of URB1418, Figure S8: MS(ESI) Spectra of URB1418, Table S1: Water Solubility Data of URB1418.
