# Supplementary material for: Synthesis and Biological Characterization of the New Glycolipid Lactose Undecylenate (URB1418)
Source: Pharmaceuticals (Basel). 2022 Apr 8;15(4):456. doi: 10.3390/ph15040456 (PMC9030338; doi:10.3390/ph15040456)
Supplement: Supplementary file 1 [file pharmaceuticals-15-00456-s001.zip › pharmaceuticals-1653513-supplementary.pdf]

# Supplementary material

## Synthesis and Biological Characterization of the New Glycolipid Lactose Undecylenate (URB1418)

Michele Verboni, Serena Benedetti, Raffaella Campana, Francesco Palma, Lucia Potenza, Maurizio Sisti, Andrea Duranti* and Simone Lucarini

[a]Department of Biomolecular Sciences, University of Urbino Carlo Bo, 61029, Urbino (PU), Italy.

* Corresponding authors.

*E-mail address*: andrea.duranti@uniurb.it (A. Duranti)

**Table of Contents**


| | | |
|---|---|---|
| 1. | Graphical Representation of $^{1}$H NMR, $^{1}$H-$^{1}$H COSY NMR, $^{13}$C NMR and MS(ESI) Spectra of Compounds **3** [Lactose Tetra Acetate (LTA) Undecylenate] and **4** (URB1418) | S2 |
| 2. | Water Solubility Data of URB1418 | S7 |

**Figure S1**. $^{1}H$ NMR Spectra of LTA Undecylenate.

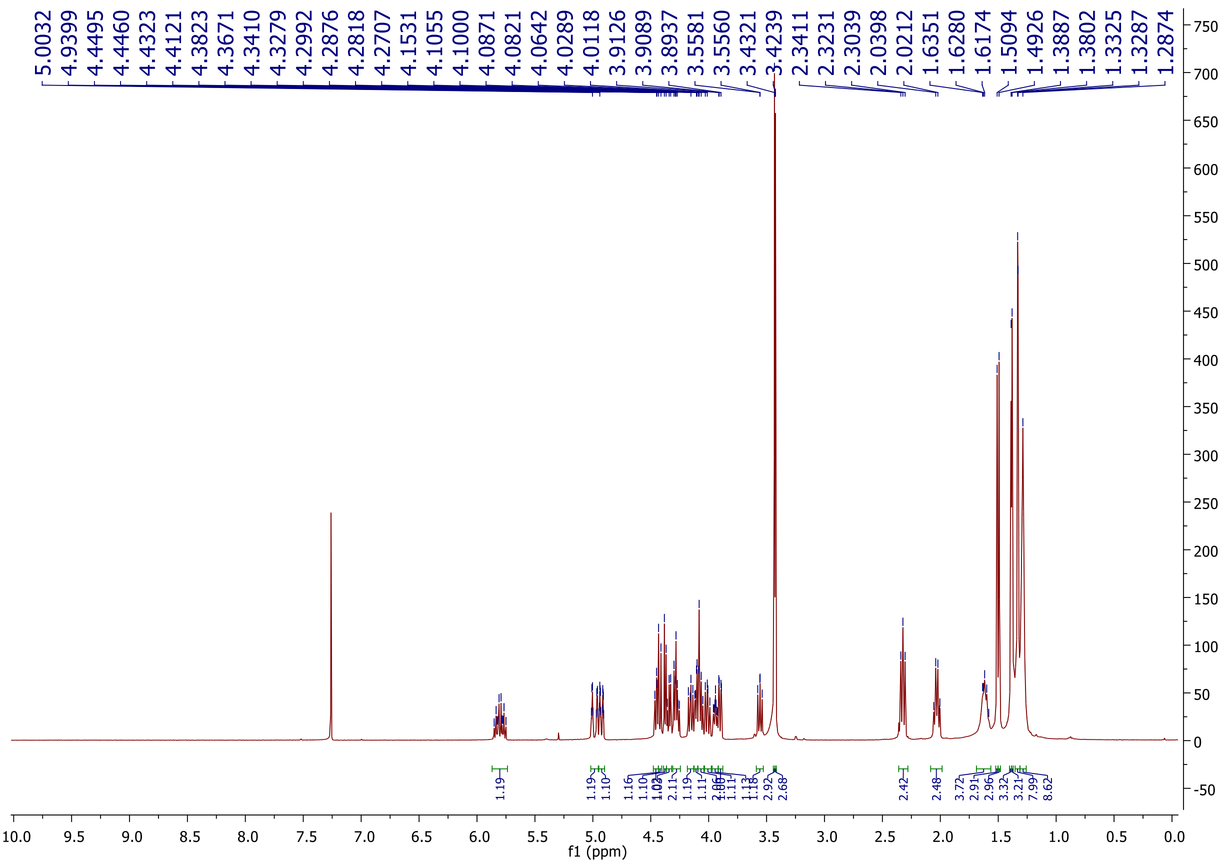


**Figure S2**. $^{1}H$-$^{1}H$ COSY NMR Spectra of LTA Undecylenate.

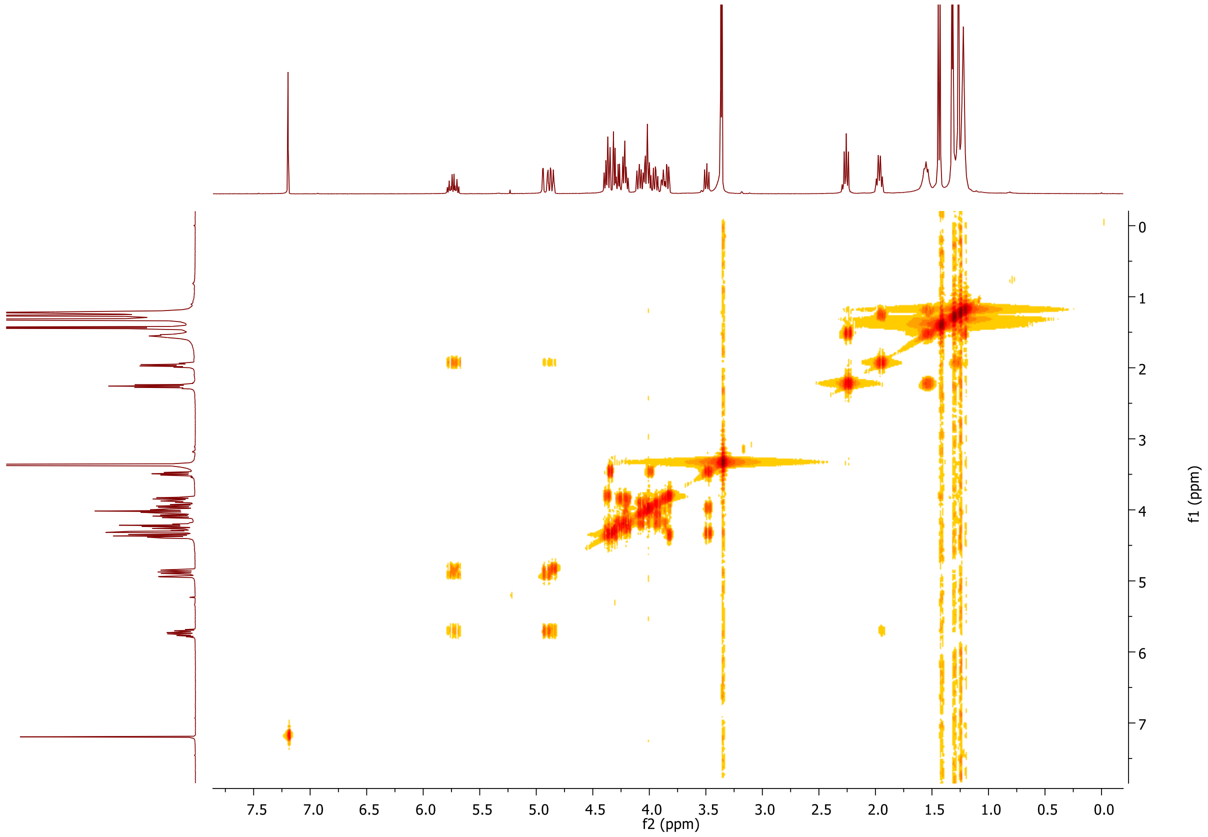

**Figure S3**. $^{13}$C NMR Spectra of LTA Undecylenate.

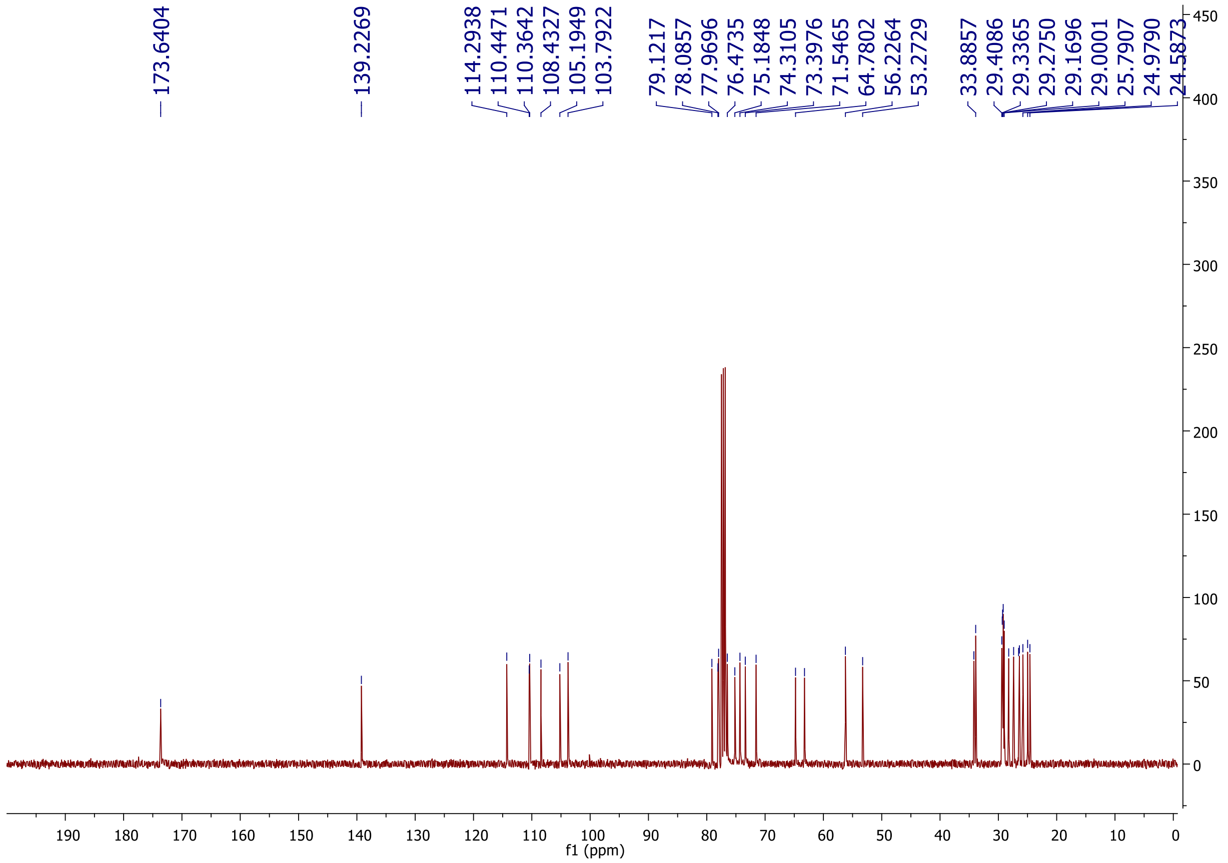

**Figure S4**. MS(ESI) Spectra of LTA Undecylenate.

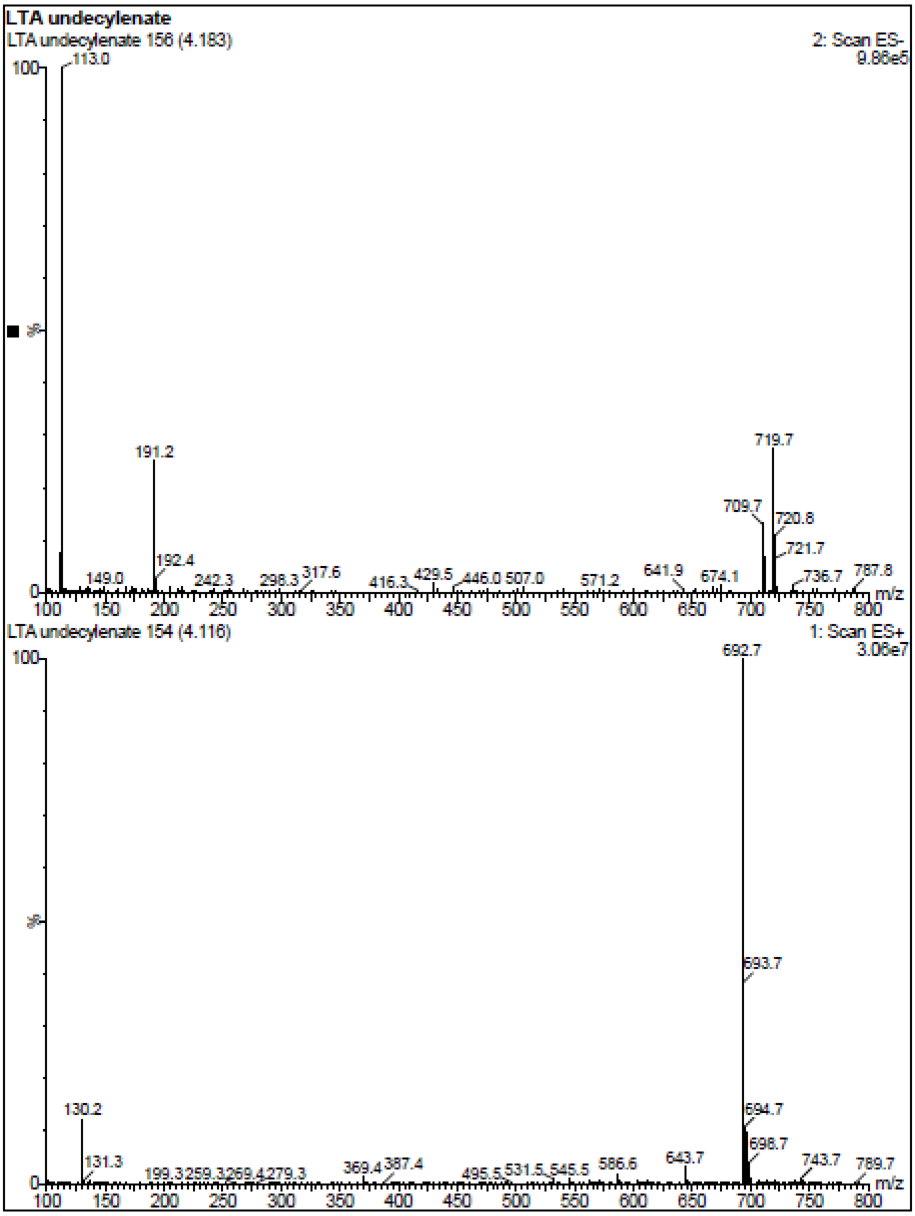

**Figure S5**. $^{1}$H NMR Spectra of URB1418.

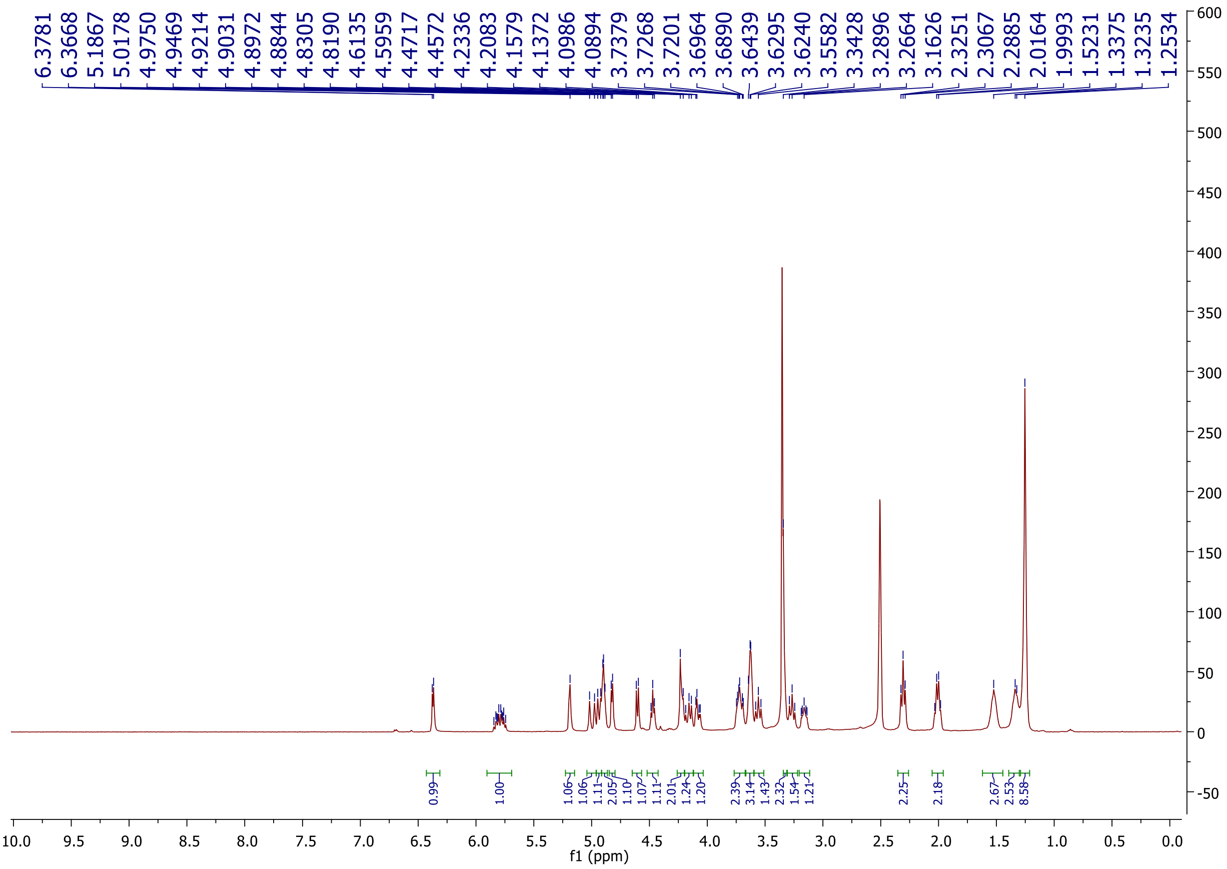


**Figure S6**. $^{1}$H-$^{1}$H COSY NMR Spectra of URB1418.

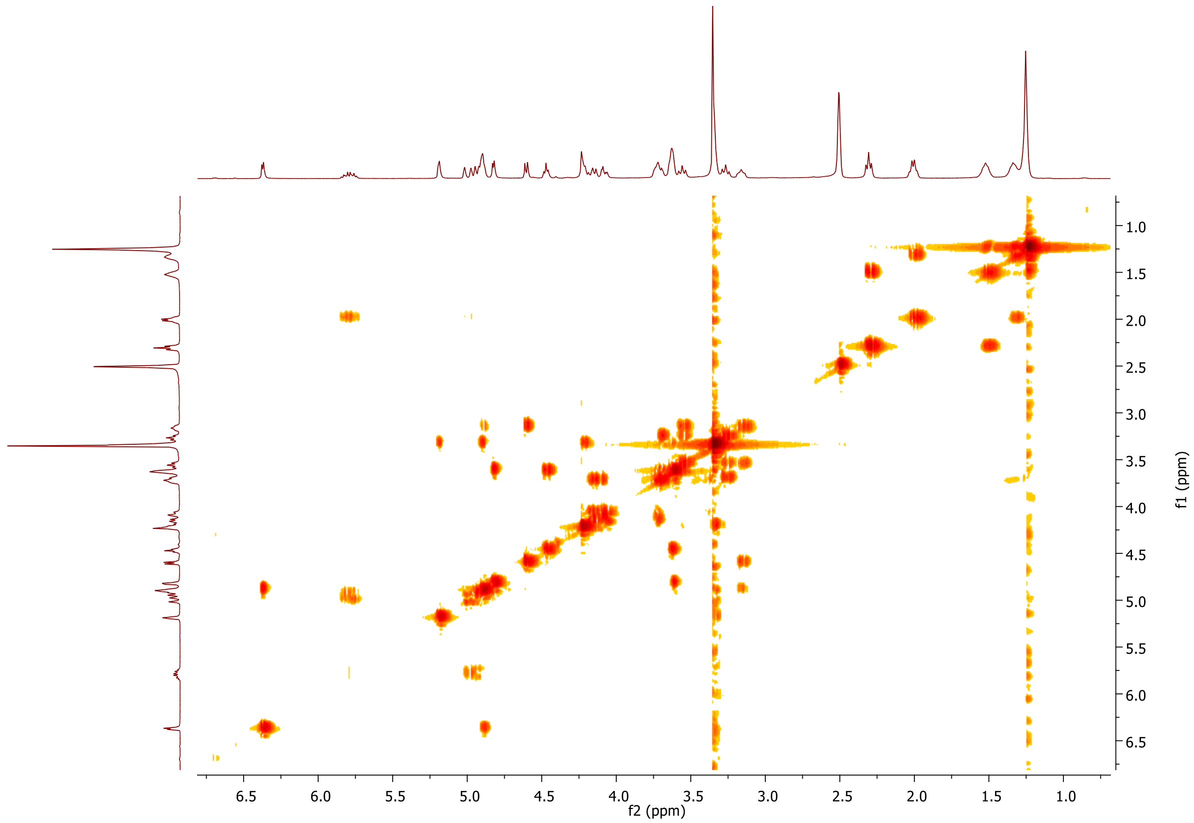

**Figure S7**. $^{13}C$ NMR Spectra of URB1418.

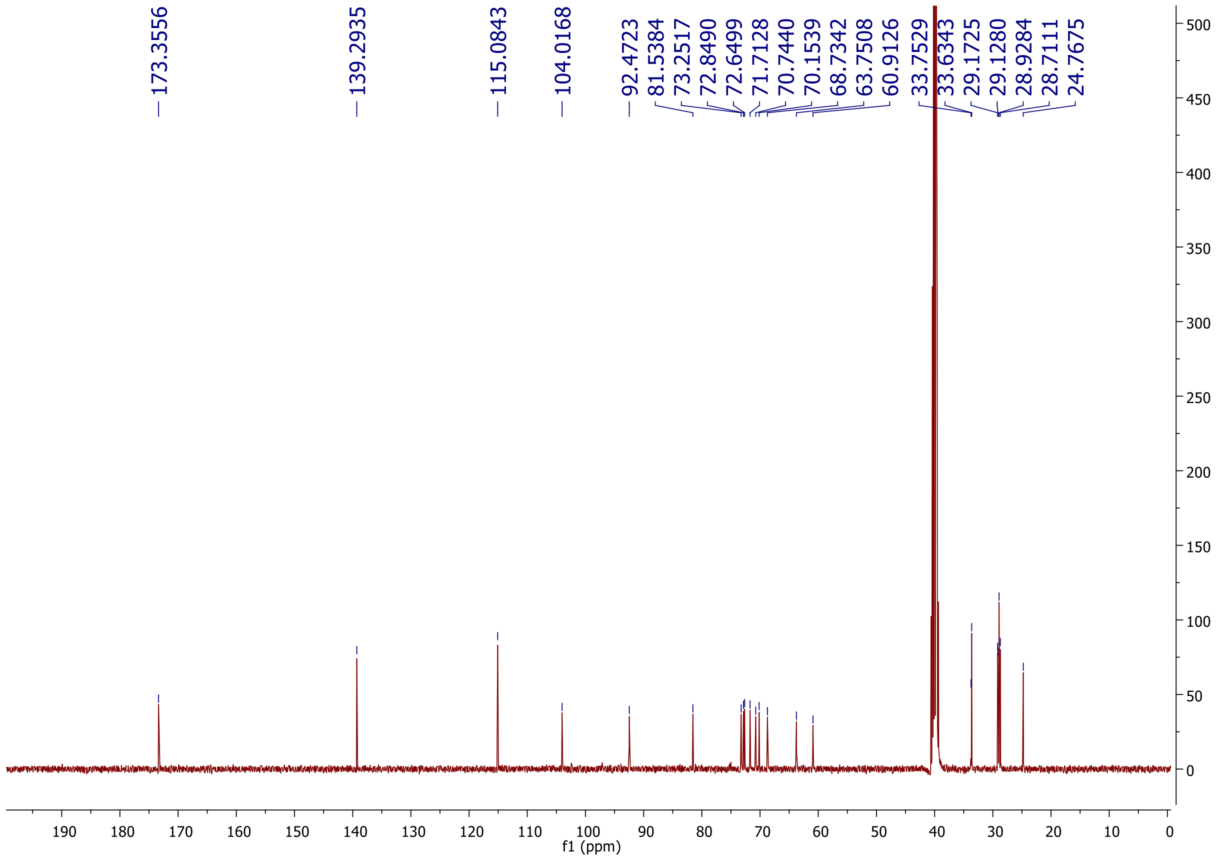

**Figure S8**. MS(ESI) Spectra of URB1418.

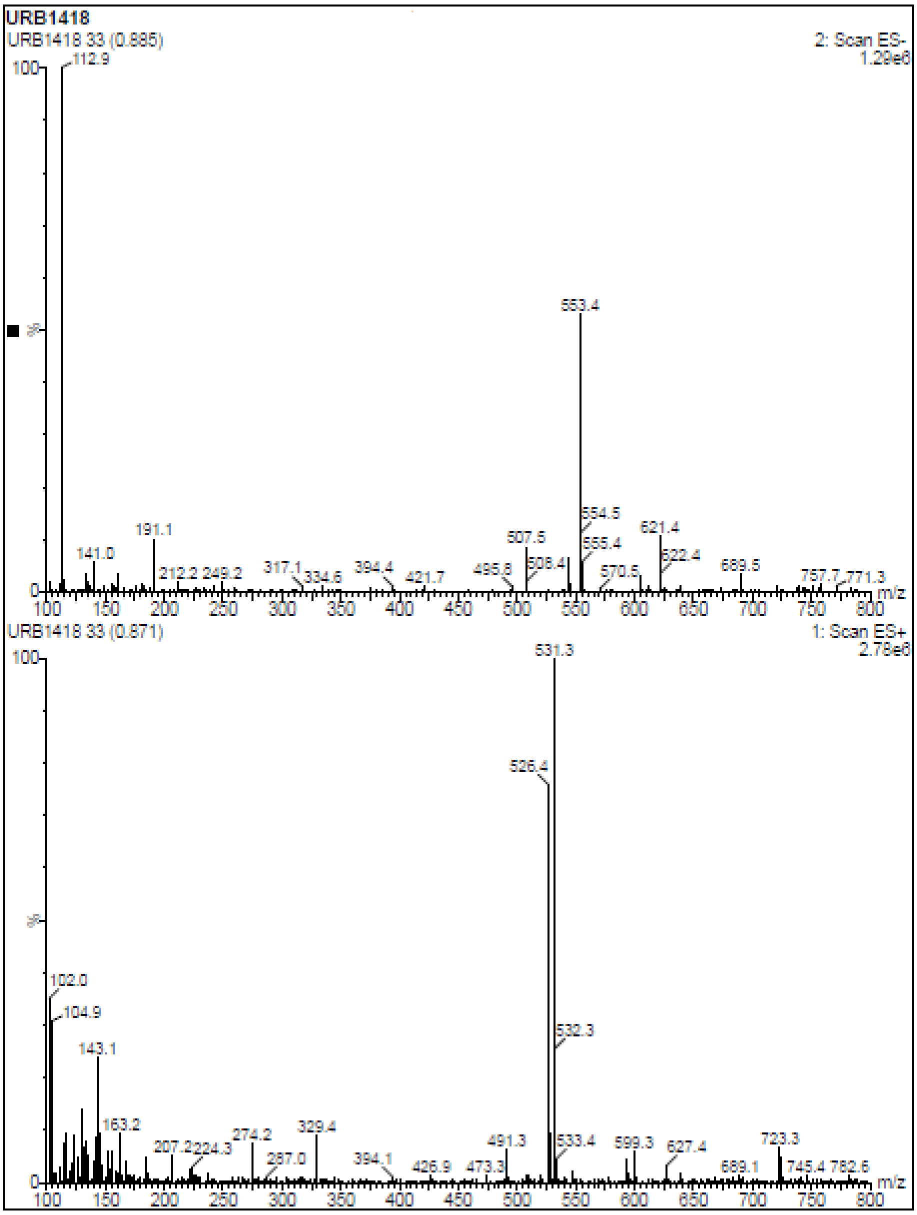

**Table S1**. Water Solubility Data of URB1418.

| Water solubility (mg/mL) | % w/v (g/100 mL) | mM (mmol/L) |
|---|---|---|
| 1.6 | 0.163 | 3.2 |
